# Association between Variants on Chromosome 4q25, 16q22 and 1q21 and Atrial Fibrillation in the Polish Population

**DOI:** 10.1371/journal.pone.0021790

**Published:** 2011-07-08

**Authors:** Marek Kiliszek, Maria Franaszczyk, Edward Kozluk, Piotr Lodzinski, Agnieszka Piatkowska, Grażyna Broda, Rafal Ploski, Grzegorz Opolski

**Affiliations:** 1 First Department of Cardiology, Medical University of Warsaw, Warsaw, Poland; 2 Department of Medical Genetics, Medical University of Warsaw, Warsaw, Poland; 3 National Institute of Cardiology, Warsaw, Poland; Mayo Clinic, United States of America

## Abstract

**Background:**

Genome-wide studies have shown that polymorphisms on chromosome 4q25, 16q22 and 1q21 correlate with atrial fibrillation (AF). However, the distribution of these polymorphisms differs significantly among populations.

**Objective:**

To test the polymorphisms on chromosome 4q25, 16q22 and 1q21 in a group of patients (pts) that underwent catheter ablation of AF.

**Methods:**

Four hundred and ten patients with AF that underwent pulmonary vein isolation were included in the study. Control group (n = 550) was taken from healthy population, matched for age, sex and presence of hypertension. All participants were genotyped for the presence of the rs2200733, rs10033464, rs17570669, rs3853445, rs6838973 (4q25), rs7193343 (16q22) and rs13376333 (1q21) polymorphisms.

**Results:**

All the polymorphisms tested (except rs17570669) correlated significantly with AF in univariate analysis (p values between 0.039 for rs7193343 and 2.7e-27 for rs2200733), with the odds ratio (OR) 0.572 and 0.617 for rs3853445 and rs6838973, respectively (protective role) and OR 1.268 to 3.52 for the other polymorphisms. All 4q25 SNPs tested but rs3853445 were independently linked with AF in multivariate logistic regression analysis. In haplotype analysis six out of nine 4q25 haplotypes were significantly linked with AF. The T allele of rs2200733 favoured increased number of episodes of AF per month (p = 0.045) and larger pulmonary vein diameter (recessive model, p = 0.032).

**Conclusions:**

Patients qualified for catheter ablation of AF have a significantly higher frequency of 4q25, 16q22 and 1q21 variants than the control group. The T allele of rs2200733 favours larger pulmonary veins and increased number of episodes of AF.

## Introduction

From the epidemiological perspective atrial fibrillation (AF) represents the most serious problem of all cardiac arrhythmias due to its high prevalence and association with increased risk of stroke, heart failure, and death [1]. The mean annual healthcare cost of a patient with AF in European countries ranges from 1000 to 3200 EUR [2]. The treatment of AF has significantly changed in the past decade due to the introduction of ablation which offers a potential curative strategy for patients with paroxysmal, persistent, or permanent atrial fibrillation [3].

Several genetic loci responsible for familial AF have been identified, such as potassium- and sodium-channel genes [4], [5], [6], but they account for only a small fraction of all AF cases. A recent genome-wide association study has identified variants on chromosome 4q25 that predispose to AF and are relatively common. In particular, two single-nucleotide polymorphisms (SNPs), rs2200733 and rs10033464, have been pointed out as markers most strongly associated with the disease. The association between AF and rs2200733/ rs10033464 has been observed in Icelandic, Swedish, USA and Chinese [7] populations as well as the population of the Framingham Heart Study and several other Western European populations [8]. Later on several other SNPs on chromosome 4q25 (rs17570669, rs3853445, rs6838973) were found, but also other loci on 16q22 (rs7193343) and 1q21 (rs13376333) [9], [10], [11]. These correlations have not been studied in the Polish population until now.

Since the 4q25 haplotype block is located about 100–200 thousand base pairs away from known genes, its functional significance is not known. The closest gene is *PITX2* encoding a transcription factor that influences heart development, especially the pulmonary veins [7]. rs7193343 is an intronic SNP located in the zinc finger homeobox 3 (ZFHX3) gene on chromosome 16q22 [10], while SNP rs13376333 is located in an intron of the calcium activated potassium channel KCNN3 gene [11].

The aim of our study was (a) to determine the association between variants on chromosome 4q25, 16q22 and 1q21 and AF in a highly selected group of Polish patients that underwent pulmonary vein isolation, and (b) to explore the putative influence of those variants on left atrium diameter (antero-posterior), pulmonary vein diameter and presence of patent foramen ovale (PFO), as well as several clinical parameters such as paroxysmal-persistent AF, coexistence of atrial flutter (AFL), AF burden (number of episodes of AF), age of onset of AF, and frequency of lone AF.

## Methods

We performed a case-control study.

### Description of the case group

The study comprised 410 consecutive patients with AF (paroxysmal or persistent) that underwent pulmonary vein isolation in the years 2006–2009. Inclusion criteria: symptomatic AF without reversible cause, unsuccessful treatment with at least one antiarrhythmic drug (group Ic or III), age below 70 years. Exclusion criteria: hyperthyroidism, significant mitral stenosis/insufficiency, left atrial dimension over 5.5 cm, severe disease with life expectancy below 1 year. Before each procedure patients underwent either transesophageal echocardiography or computer tomography of the left atrium.

The study was approved by the Bioethics Committee of the Medical University of Warsaw and all patients gave written informed consent.

The following clinical parameters were recorded: presence of hypertension, coronary disease, heart failure, diabetes, history of hyperthyroidism. Patients were also asked about the year of the onset of AF and mean number of episodes of AF per month (within the last 6 months). Presence of concomitant atrial flutter (AFL) was defined as at least one episode of AFL with a typical ECG pattern. Definitions of paroxysmal, persistent and lone AF were according to the European guidelines [12]. Diagnosis of patent foramen ovale (PFO) was based on transesophageal echocardiography (TEE). Measurements of pulmonary vein diameters were based on TEE, only examinations made by the same operator (n = 209) were included in the subanalysis.

### Description of the control group

DNA samples from controls were obtained from a bank of samples collected during a recent Polish population-wide health survey WOBASZ (Wieloośrodkowe ogólnopolskie badanie stanu zdrowia ludności, National Polish Health Survey [13]). The control group (n = 550) included subjects from background population that did not have a history of atrial fibrillation or cardiac arrhythmia, and was matched for age, sex and presence of hypertension with the patients. The study of the WOBASZ cohort was approved by The Medical Ethics Committee of National Institute of Cardiology in Warsaw. Each participant gave two written consents: for use of questionnaire data and for taking blood sample for DNA isolation and genetic analyses.

### Genotyping

Genomic DNA was isolated from peripheral blood by salting out. Genotyping of SNPs: rs2200733, rs10033464, rs17570669, rs3853445, rs6838973, rs7193343 and rs13376333 was performed using Taqman Assays (C_16158671_10, Custom TaqMan SNP Genotyping Assay, C__33254659_10, C__1176985_10, C__29128132_20, C__29343982_10 and C__2745708_10 respectively) (Applied Biosystems, Foster City, California, USA). Assays were performed according to the manufacturer's instructions using a 7500 Real-Time PCR System (Applied Biosystems).

### Statystical analysis

Continuous variables are presented as mean ±SD (standard deviation) or median (1–3 quartile), depending on variable distribution. Categorical variables are presented as frequencies. The Chi2 or Fisher exact tests were used to test deviations of the genotype distribution from Hardy–Weinberg equilibrium and to compare allele and genotype frequencies between groups. Effects of genotypes on continuous parameters were analyzed by one-way ANOVA or relevant non-parametric tests (Kruskal-Wallis). Comparison of diameters of pulmonary veins in the recessive model, due to significant deviations from normal distribution, was performed with Mann-Whitney U test. Multivariate logistic regression was performed to assess independent significance of all 4q25 polymorphisms. Haplotype analysis was performed with PLINK software [14].

Sample size calculation (post-hoc) was performed using top 4q25 polymorphisms (rs2200733 and rs10033464). Based on previous results we assumed polymorphic allele frequency of rs10033464 equal to 0.08 for controls and 0.12 for cases, and for rs2200733 0.11 and 0.17, respectively [7]. At alpha = 0.05 to achieve 80% power with the controls/cases ratio = 1.5, the minimal number of cases and controls was 365 and 545 for rs10033464, and 225 and 335 for rs2200733.

## Results

The characteristics of the patients and controls is shown in table 1. There were no statistically significant differences between the two groups with respect to age, sex and presence of hypertension (table 1). Among both patients and controls the genotype distribution did not deviate from Hardy–Weinberg equilibrium (from 0.035 and 0.045 for rs7193343 and rs6838973 control group respectively [we considered it non significant after Bonferroni correction], to 1.00 in rs13376333 cases group).

**Table 1:** Characteristics of study and control groups.

	cases	controls
**Age** *	53.3 (11.3)	53.1 (10.4)
**Women**	128 (31.2)	169 (30.7)
**Hypertension**	234 (57.1)	310 (56.4)
**Diabetes**	35 (8.5)	ND
**Coronary disease**	40 (9.8)	0 (0)
**Heart failure**	6 (1.5)	0 (0)
**History of hyperthyroidism**	85 (20.7)	ND
**Current smoker**	17 (4.1)	187 (34)
**Hyperlipidemia**	86 (21.5)	ND
**Family history**	13 (3.2)	NA
**Age of AF diagnosis** *	46.7 (10.7)	NA

Data presented as: n (%).

*mean (SD).

SD – standard deviation.

ND – no data; NA – not applicable.

For all SNPs with the exception of rs17570669 there was a statistically significant difference in polymorphic allele frequencies between cases and controls (table 2). Pairwise analyses among the 4q25 SNPs showed a weak LD (r^2^< = 0.1) except for rs3853445–rs6838973 (r^2^∼0.4, both among cases and controls, table 3). In multivariate logistic regression analysis, rs6838973 was significantly associated with AF (OR 0.608; 95% CI 0.475–0.778; p = 0.000079) whereas rs3853445 was not (OR = 0.9, p = 0.43). To further study independence of effects of 4q25 SNPs on AF risk a multivariate logistic regression model was built including rs2200733, rs10033464, rs17570669 and rs6838973. All four SNPs were found to be associated with AF: in the case of rs2200733 and rs10033464 the effects were in the direction of increased risk (OR = 3.743, p = 1.21E-20 and OR = 2.362 and P = 2.12E-06, respectively) whereas for rs17570669 and rs6838973 protective effects were observed (OR = 0.608, p = 0.046 and OR = 0.661, p = 0.00033, respectively, table 4).

**Table 2:** Genotype distribution of analyzed SNPs among patients and controls.

chromosome	SNP	Minor allele	Patients	Controls	OR	CI	p
**4q25**	**rs2200733**	T	39/173/177	12/109/425	3.369	2.633–4.31	4.526e-22
**4q25**	**rs10033464**	T	3/87/295	2/79/460	1.695	1.23–2.335	0.001253
**4q25**	**rs17570669**	T	0/46/364	5/69/477	0.7725	0.532–1.122	0.1751
**4q25**	**rs3853445**	C	16/122/272	44/215/296	0.6227	0.5003–0.7751	2.232e-5
**4q25**	**rs6838973**	T	42/185/172	131/241/158	0.575	0.4756–0.695	1.074e-8
**1q21**	**rs13376333**	T	45/172/159	53/200/280	1.328	1.087–1.622	0.0054
**16q22**	**rs7193343**	T	27/128/230	28/148/344	1.268	1.012–1.590	0.039

Number of polymorphic homozygotes/polymorphic heterozygotes/wild types are given. OR – odds ratio. CI – 95% confidence interval (calculated by univariate logistic regression assuming additive model).

**Table 3:** Pairwise linkage disequilibrium (LD) scores (r2) among 4q25 SNPs.

Patients				
	rs2200733	rs10033464	rs17570669	rs3853445
rs10033464	0.05			
rs17570669	0.03	0.02		
rs3853445	0.04	0.07	0.03	
rs6838973	0.03	0.01	0.13	0.39

Controls				
rs10033464	0.01			
rs17570669	0.08	0.01		
rs3853445	0.0000	0.05	0.10	
rs6838973	0.003	0.03	0.08	0.42

**Table 4:** Multivariate logistic regression of 4q25 SNPs with independent effects on AF risk.

	OR	CI	p
**rs2200733**	3.743	2.836–4.941	1.21e-20
**rs10033464**	2.362	1.656–3.370	2.12e-6
**rs17570669**	0.608	0.373–0.991	0.046
**rs6838973**	0.661	0.528–0.829	0.00033

OR – odds ratio; CI – confidence interval.

When analysis of haplotypes was performed a highly statistically significant overall association was observed (P = 1.86E-32, omnibus test of haplotype association). The strongest effect was conferred by the TGAC haplotype which had a frequency of 0.257 among cases vs. 0.074 among controls, conferred a relatively high risk for AF (OR = 4.33) and was highly significantly associated with the disease (p = 1.187E-27, table 5).

**Table 5:** Haplotypes found for 4q25 SNPs with independent effects on AF risk (i.e. rs2200733-rs10033464-rs17570669-rs6838973  haplotypes).

Haplotype	Patients	Controls	OR	p
TGTT	0.035	0.031	1.14	0.6266
CGTT	0.017	0.039	0.44	0.006895
CTAT	0.061	0.052	1.17	0.4411
TGAT	0.028	0.014	1.96	0.04009
CGAT	0.199	0.337	0.49	5.08e-11
CTAC	0.059	0.025	2.49	0.000147
TGAC	0.257	0.074	4.33	1.18e-27
CGAC	0.345	0.428	0.70	0.000273
TGTT	0.035	0.031	1.14	0.6266

There was a statistically significant correlation between rs2200733 and the number of AF episodes per month (p = 0.045, one way ANOVA). The mean number of AF episodes (95% CI) was 7.3 (5.4–9.1), 8.8 (6.8–10.8) and 12.9 (8.8–16.3) for patients with the CC, CT and TT genotypes, respectively. The haplotype with the strongest link with AF (TGAC, see table 5) was the only one significantly linked with the number of AF episodes (beta = 3.04, p = 0.0048).

We also observed a statistically significant correlation between the rs2200733 genotypes and pulmonary vein diameter in a recessive model (p = 0.032, Mann-Whitney U test for mean value of all pulmonary veins). Age or sex did not affect significantly the pulmonary vein diameter. The correlation was significant for the left inferior pulmonary vein whereas there was a trend for the left and right superior and no effect for the right inferior pulmonary vein (table 6). None of the other polymorphisms (rs10033464, rs17570669, rs6838973, rs3853445, rs7193343, rs13376333) correlated with the number of AF episodes nor with diameter of pulmonary veins (data not shown).

**Table 6:** Correlation of rs2200733 genotype (CC and CT vs. TT) with diameter of pulmonary veins (in millimetres).

Pulmonary veins	CC and CT	TT	p
**Left superior**	16 (14–18)	17.5 (15–19)	0.127
**Left inferior**	16 (14–18)	17 (16.0–19)	0.012
**Right superior**	17 (15–19)	18 (16–20)	0.101
**Right inferior**	17 (15–19)	17.3 (16–20)	0.330
**Mean value**	16.25 (15–18.25)	18 (16.75–19.5)	0.032

For calculation a recessive effect was assumed.

Data presented as median (1^st^–3^rd^ quartile).

We did not see any correlation of the polymorphsims tested with concomitant diseases (hypertension, diabetes, coronary disease, heart failure) or with the frequency of lone AF, persistent AF, AFL coexistence, age of diagnosis of AF, or left atrial diameter (data not shown).

## Discussion

Our results provide a strong confirmation of the association between AF and 4q25, 16q22 and 1q21 variants, in particular rs2200733 for which the statistical significance of the observed association (p = 2.7×10^−27^) exceeded the threshold used for genome-wide studies. We also provide the first confirmation of the results recently reported by Lubitz et al. [9] who found that rs3853445 and rs6838973 were both associated with AF but most likely were markers of a single primarily associated variant. Our data suggest that rs6838973 might be in a stronger LD with this primary variant as in our data it accounted for the association of rs3853445. We also confirmed the observation [9] that rs17570669, while not associated with AF in a univariate analysis, has a protective effect in an analysis adjusted for other markers in the 4q25 region. In contrast to Lubitz et al. [9] but in agreement with earlier results [7], [8] we observed a strong independent susceptibility conferring effect of rs10033464. The association of SNPs on 16q22 and 1q21 with AF is the first confirmation of the results by Gudbjartsson [10] and Ellinor [11].

Our AF group was homogenous and well described. It comprised consecutive patients, thus reflecting everyday clinical practice. The control group was matched with the patients regarding region of birth, age, sex and presence of hypertension. The frequency of the polymorphic alleles in the control group was comparable to those found in other studies [7], [8].

Comparing with the previous studies in European populations, the frequency of the polymorphic rs2200733 allele in the Polish AF group was relatively high. A German group with lone AF [8] and another German group that underwent catheter ablation [15] had lower frequency of the T allele (0.27). There are three major differences between our group and the non-ablation groups – (i) our patients were highly symptomatic, (ii) had a high burden of AF (many patients had paroxysms of AF almost every day) and (iii) were drug-resistant. In particular, our results support the importance of the burden of AF – patients with the T allele of rs2200733 had more paroxysms of AF per month than did patients with the C allele, which could explain why such patients are overrepresented in the group qualified for catheter ablation. That observation was confirmed in haplotype analysis – the TGAC haplotype (see table 5) with the strongest relationship with AF was the only one linked with the number of AF episodes. The frequency of three other polymorphisms form the 4q25 region was comparable to that in other AF populations of European ancestry [7], [8], [10]. In a logistic regression analysis we found that all 4q25 polymorphisms tested were independently linked with AF. We identified nine 4q25 haplotypes, of which six were significantly linked with AF.

We also confirmed other independent markers of AF: rs7193343 on chromosome 16q22 and rs13376333 (1q21). Although only about 30% of our group had lone AF, the frequency of rs13376333 was comparable to that in a population with lone AF [11]. The frequency of rs7193343 was comparable to that found in a previous study [10].

We observed that patients with the T allele of rs2200733 had a larger diameter of pulmonary veins, in particular when the genetic effect was modeled as recessive. That could be related to the earlier observation that patients with the T allele have more recurrences after catheter ablation [15], although it has been shown that the diameter of pulmonary veins has no impact on the outcome of catheter ablation [16]. The problem needs further studies.

The mechanisms of the effect of the 4q25 genetic variants on AF is not clear. The nearest gene is PITX2 (Paired-like homeodomain transcription factor 2), important for determining the left–right asymmetry and for the differentiation of the left atrium [17]. PITX2c-deficient mice do not develop pulmonary myocardial sleeves [18] – clinical studies have demonstrated that those sleeves generate ectopic beats that play a substantial role in initiating and maintaining atrial fibrillation [19]. Electrical isolation of those sleeves in pulmonary veins is a cornerstone for most AF ablation procedures [20]. The 1q21 locus is at the KCNN3 gene which codes for a member of a family of calcium-activated potassium channels [11]. It has been shown on an animal model that KCNN plays a role in atrial action potential duration [21] – shortening of action potential duration is an important mechanism for the development and maintenance of the AF. rs7193343 is an intronic SNP located in the zinc finger homeobox 3 (ZFHX3) gene (chromosome 16q22) [10]. The gene may play a role in regulation of growth and differentiation of neuronal and skeletal muscle [22], but its relation to AF is unknown.

### Limitations of the study

The main limitation of the study is connected with the high number of hypotheses tested in the exploration of novel genotype –phenotype correlations. Therefore all the observations regarding new associations/correlations should be verified in adequately powered dedicated studies.

### Conclusions

Polish patients qualified for catheter ablation due to AF have a significantly higher frequency of 4q25 (especially rs2200733), 16q22 and 1q21 variants than the control group. The T allele of rs2200733 seems to favour larger pulmonary veins and a higher number of episodes of AF but it should be verified in future studies.
